# Brain structural changes in girls with idiopathic central precocious puberty: a voxel-based morphometry and surface-based morphometry analysis

**DOI:** 10.3389/fendo.2025.1643660

**Published:** 2025-10-07

**Authors:** Yun Zhang, Lu Tian, Xiaojian Wang

**Affiliations:** ^1^ Department of Radiology, Shenzhen Longhua Maternity and Child Healthcare Hospital, Shenzhen, China; ^2^ Department of Radiology, Children’s Hospital of Chongqing Medical University, Chongqing, China; ^3^ Department of Radiology, The First Affiliated Hospital of Chongqing Medical and Pharmaceutical College, Chongqing, China

**Keywords:** idiopathic central precocious puberty, girls, voxel-based morphometry, surface-based morphometry, ICPP

## Abstract

**Objectives:**

This study aimed to investigate brain structural alterations in girls with idiopathic central precocious puberty (ICPP) using voxel-based morphometry (VBM) and surface-based morphometry (SBM), and to explore associations between these changes and clinical indicators to elucidate the impact of ICPP on brain structure and uncover its underlying mechanisms.

**Materials and methods:**

Fifty-two girls aged 6–8 years with ICPP and 50 age-matched non-ICPP girls underwent 3.0 T MRI T1-weighted imaging. Structural data were analyzed using SPM12 and CAT12. A two-sample t-test assessed differences in global and regional brain volumes and cortical thickness. Correlation analyses explored relationships between structural alterations and clinical indicators.

**Results:**

(1) The ICPP group showed increased total intracranial volume, white matter volume, and cerebrospinal fluid volume, but decreased gray matter volume (*p* < 0.05). (2) VBM revealed reduced gray matter volume in the right precentral gyrus, bilateral amygdala, bilateral inferior temporal gyrus, and bilateral putamen (FDR-corrected, *p* < 0.05). (3) SBM showed cortical thinning in the right precentral gyrus (FDR-corrected, *p* < 0.05). (4) Gray matter volume in the inferior temporal gyrus and right precentral gyrus, and cortical thickness in the right precentral gyrus, were negatively correlated with peak LH levels (FDR-corrected, *p* < 0.05).

**Conclusions:**

VBM and SBM effectively identify brain structural changes in ICPP girls, particularly in regions related to motor control, emotion, and cognition. These alterations, linked to hormonal levels, may serve as imaging biomarkers and offer insights into the neurobiological impact of ICPP.

## 1 Introduction

Idiopathic central precocious puberty (ICPP) refers to the premature activation of the hypothalamic-pituitary-gonadal (HPG) axis without identifiable organic causes, leading to the early development of secondary sexual characteristics—before age 8 in girls and before age 9 in boys (1, 2). Studies have shown that the incidence of ICPP is on the rise, with a significantly higher prevalence in females—approximately 10 to 20 times that in males (3–7). Globally, incidence rates vary markedly across geographic regions, ranging from 0.217 to 26.28 cases per 10,000 girls and from 0.02 to 0.9 cases per 10,000 boys (3–6). For the Chinese population, a school-based study (covering 12,876 children aged 6–12 years) reported a prevalence of precocious puberty (including ICPP) of 0.43% among girls, which aligns with the demographic characteristics of our participants (Chinese girls aged 6–8 years) (7). These epidemiological data are primarily derived from national patient registries, national insurance claims databases, and statistics from tertiary medical centers (8, 9).

Clinically, ICPP has sex-specific manifestations: girls initially show breast development, then pubic/axillary hair growth; boys first have testicular/penile enlargement, followed by pubic/axillary hair growth and voice deepening (10). These manifestations, though consistent across populations, underlie profound physiological, psychological, and social impacts of ICPP—including premature epiphyseal closure (risking reduced adult height), early sex hormone exposure (potentially increasing long-term cancer risk), and psychosocial challenges like anxiety or peer exclusion (1, 10, 11).

The brain plays a central role in ICPP by regulating the secretion of gonadal hormones (8, 9). As a result, the relationship between brain and ICPP has become a key focus of research. Clinically, children presenting with signs of precocious puberty are generally assessed using conventional cranial magnetic resonance imaging (MRI) to rule out intracranial anomalies such as hypothalamic hamartomas or suprasellar arachnoid cysts (12). In the majority of CPP cases, however, no obvious structural abnormalities are found in the central nervous system, leading to a diagnosis of ICPP (12).With the advancement of neuroimaging technologies, structural MRI (sMRI) has become a valuable tool for investigating brain development mechanisms. In particular, voxel-based morphometry (VBM) and surface-based morphometry (SBM) enable precise evaluation of brain microstructures by assessing gray matter volume and cortical thickness, respectively (13, 14). These methods have facilitated the identification of hormone-related structural brain changes. Accumulating evidence suggests that ICPP is associated with alterations in brain structure. For example, Yoshii et al. (15) analyzed 15 girls with ICPP and 13 age-matched controls using SBM, reporting increased cortical thickness in the right precuneus in the ICPP group. Yang et al. (16) investigated 28 ICPP girls and 37 non-ICPP girls via SBM, finding cortical thinning in the right middle frontal gyrus (negatively correlated with estradiol levels). Chen et al. (17) compared 22 ICPP girls with 20 girls with partial precocious puberty (PPP) using VBM, observing reduced gray matter volume in the left insula and fusiform gyrus in the ICPP group (with left insula volume negatively correlated with peak FSH levels).”These findings suggest that sex hormones may influence the structural remodeling of brain regions involved in emotional and cognitive regulation, underscoring the importance of monitoring the psychological health of girls with ICPP. Despite these advances, current research on brain structure in ICPP faces several limitations, including a limited number of studies, small sample sizes, reliance on single imaging modalities, and inconsistent findings across studies. In light of these issues, further in-depth research is needed to gain a more comprehensive understanding of how ICPP affects brain structure.

This study had two primary objectives: (1) To investigate brain structural changes in ICPP girls (vs. age-matched non-ICPP girls) using VBM and SBM; (2) To explore associations between these brain changes and key clinical indicators (e.g., peak LH, bone age, ovarian volume). The secondary objective was to elucidate ICPP’s neurobiological impact on brain development and the mechanisms linking HPG axis activation to brain remodeling. This is the first study to apply both VBM and SBM in this population, aiming for a comprehensive view of ICPP-related brain alterations.

## 2 Methods

**Figure 1:**
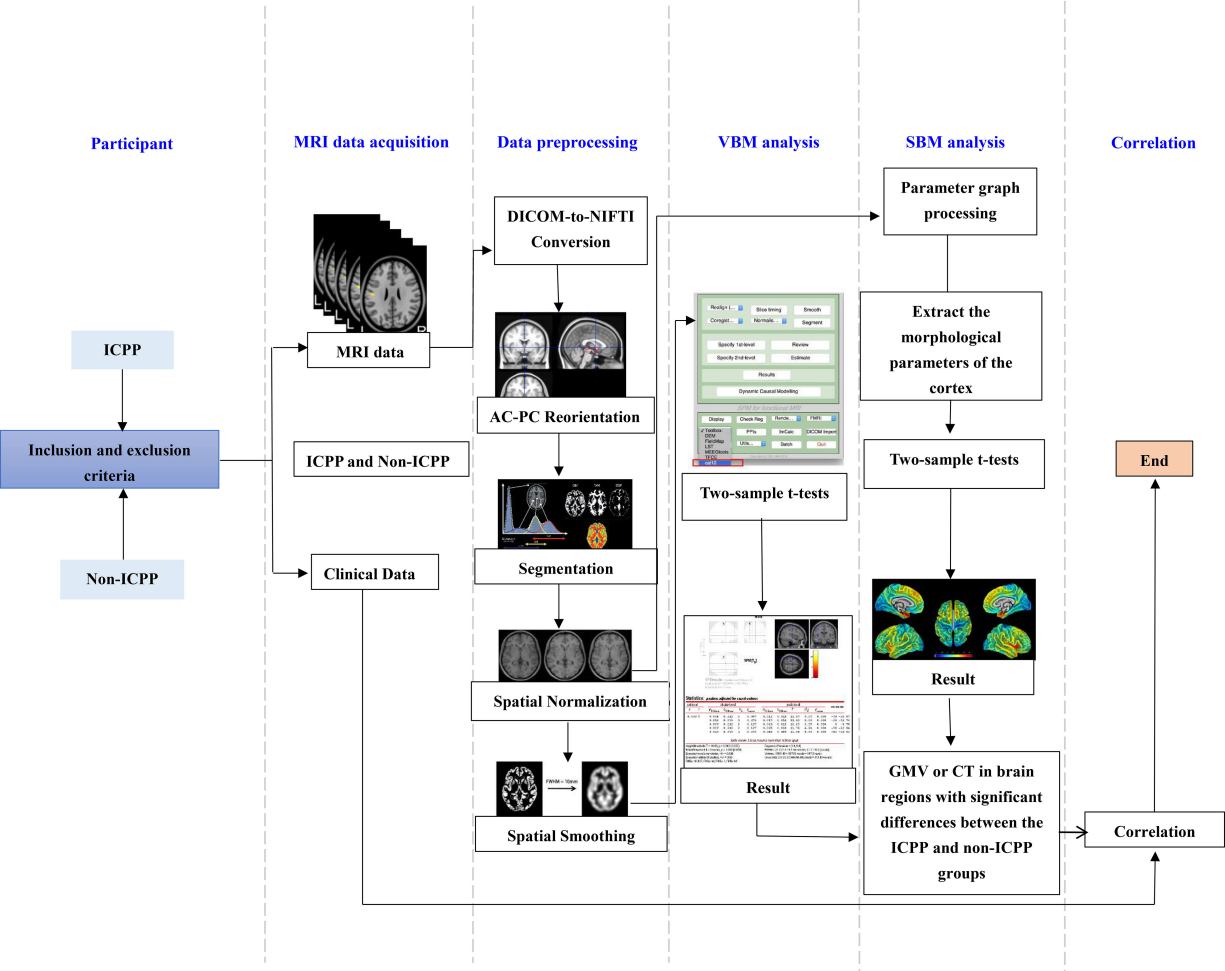
Flow chart of VBM and SBM analysis. ICPP, idiopathic central precocious puberty; non-ICPP, non-idiopathic central precocious puberty; MRI, magnetic resonance imaging; AC-PC, anterior commissure-posterior commissure; VBM, voxel-based morphometry; SBM, surface-based morphometry; GMV, gray matter volume; CT, cortical thickness.

The overall flowchart of research method is shown in **Figure 1**.

### 2.1 Participants

**Figure 2:**
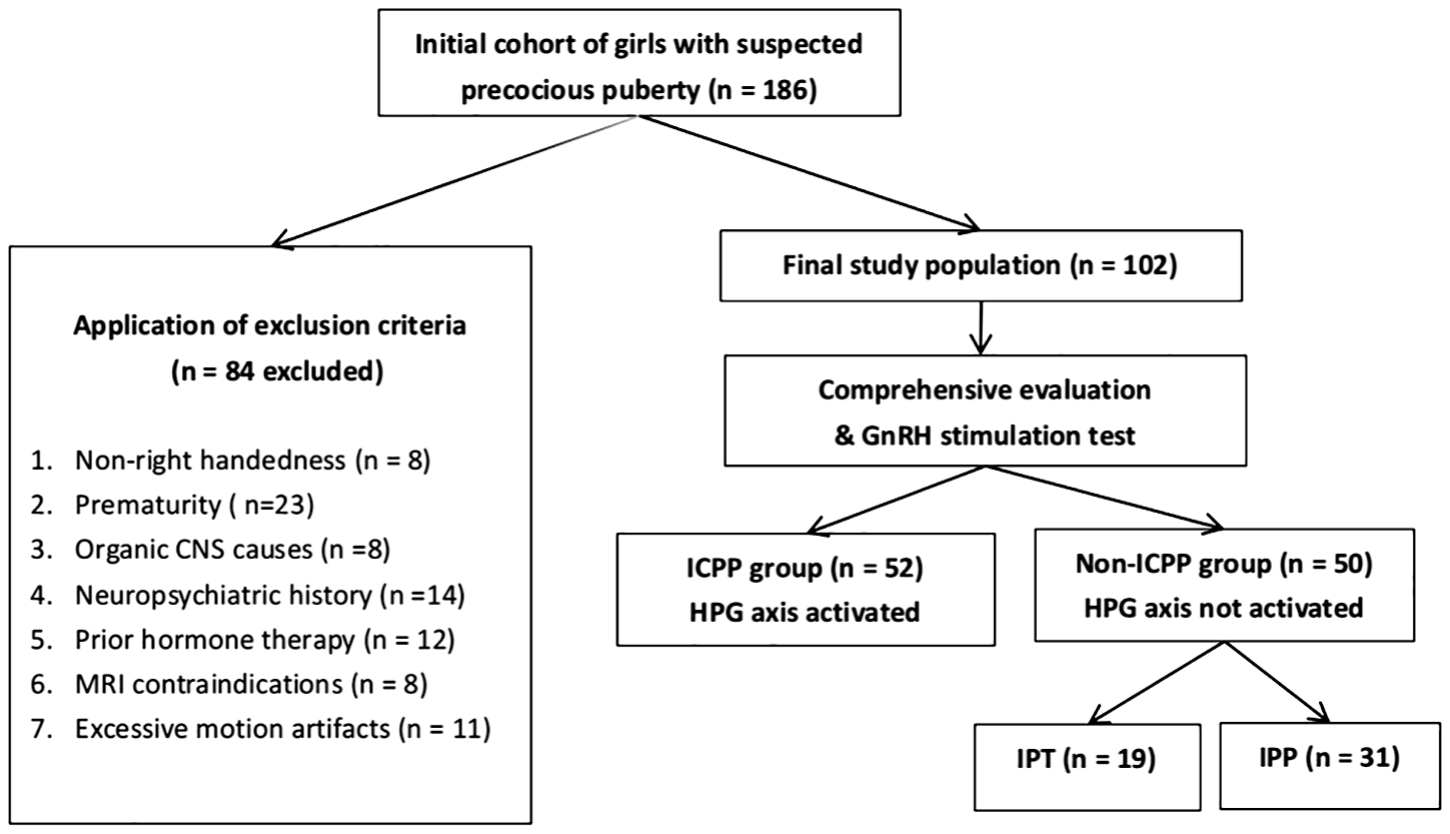
Flowchart of participant screening. ICPP, idiopathic central precocious puberty; CNS, central nervous system; GnRH, gonadotropin-releasing hormone; HPG, hypothalamic-pituitary-gonadal; IPT, isolated premature thelarche; IPP, isolated premature pubarche.

This study included 102 female children (aged 6–8 years) diagnosed with precocious puberty who presented to the endocrinology department of our hospital between January 2023 and December 2024 with clinical manifestations of secondary sexual characteristics, such as breast development or vaginal bleeding. Patients were classified through comprehensive evaluation including the gold-standard GnRH stimulation test, yielding 52 ICPP cases (mean age 6.56 ± 1.06 years) and 50 matched non-ICPP controls (mean age 6.64 ± 1.21 years). The inclusion criteria for the ICPP group were as follows: (1) bone age exceeding chronological age by at least one year; (2) breast development at Tanner stage ≥2; (3) body mass index (BMI) within the 25th to 85th percentile for age and sex; (4) normal brain and pituitary MRI findings; and (5) GnRH stimulation test results confirming HPG axis activation. The GnRH stimulation test was performed between 8:00 and 9:00 AM. Patients received an intravenous injection of a GnRH analogue (GnRHa, gonadorelin) at a dose of 2.5 μg/kg (maximum dose: 100 μg). Serum luteinizing hormone (LH) and follicle-stimulating hormone (FSH) levels were measured at 0, 30, 60, and 90 min post-injection using chemiluminescence immunoassay. Activation of the HPG axis, confirming ICPP, was defined as a peak LH level ≥5.0 U/L and a peak LH/FSH ratio ≥0.6 (18, 19). For those who did not meet this criterion, they were diagnosed with GnRH-independent precocious puberty, referred to as non-ICPP within this study. The inclusion criteria for the non-ICPP group mirrored those for ICPP, except that the GnRH stimulation test results indicated no activation of the HPG axis. Both groups excluded subjects with: non-right handedness, prematurity, organic CNS causes, neuropsychiatric history, prior hormone therapy, MRI contraindications, or excessive motion artifacts. As detailed in **Figure 2** (participant screening flowchart).

### 2.2 Clinical data

The clinical data collection protocol was developed through systematic literature review and consultations with board-certified pediatric endocrinologists. Collected indicators were systematically categorized into four aspects: general measures, clinical measures, laboratory indicators, and imaging features. General and clinical measures included chronological age, standing height, body weight, body mass index (BMI), breast development and vaginal bleeding. Laboratory indicators comprised baseline and stimulated serum concentrations of gonadotropins (FSH and LH) alongside estradiol levels through standardized immunoassays. For imaging evaluations, transabdominal pelvic ultrasonography was performed to obtain triplanar measurements (longitudinal, anteroposterior, and transverse diameters) of uterine and ovarian structures. Volume calculations employed the prolate ellipsoid formula (20): V = 0.523 × length × width × thickness.

Skeletal age was assessed via left hand-wrist radiography (TW2 system (21),) by two radiologists with ≥5 years of pediatric imaging experience (specializing in skeletal age). For 3 cases with inconsistent results, a third radiologist (10 years of pediatric imaging experience) resolved discrepancies, ensuring accurate skeletal age measurements.

The hamilton anxiety scale (HAMA) was administered to evaluate anxiety symptoms in children on the day of their MRI scan; its total scores range from 0 to 56, with scores ≥ 14 indicating significant anxiety symptoms, 7–13 indicating mild anxiety, and < 7 indicating no anxiety (22). In addition, all participants underwent assessment using the Chinese version of the wechsler intelligence scale for Children (WISC-CR) prior to MRI scanning to screen for intellectual impairment; its full-scale IQ scores range from 40 to 160, with scores ≥ 85 classified as “normal intelligence,” 70–84 as “borderline intelligence,” and < 70 as “intellectual impairment” (23). Caregivers (parents or legal guardians) were also asked to complete the child behavior checklist (CBCL), a standardized tool used to assess behavioral and emotional problems in children (24)—and its total scores range from 0 to 100, with higher scores indicating more frequent behavioral/emotional problems (e.g., aggression, withdrawal); scores > 60 are considered “clinically significant”.

### 2.3 MRI data acquisition

All MRI examinations was performed using a Philips Achieva 3.0T superconducting MRI system equipped with an 8-channel phased-array head coil for signal reception. Prior to scanning, all patients were instructed to remove any ferromagnetic items, including jewelry, mobile phones, coins, watches, and keys, to ensure both imaging safety and optimal image quality. Subjects were positioned supine with their heads immobilized within the coil using manufacturer-supplied foam pads and straps to minimize motion artifacts. Earplugs were provided to reduce discomfort caused by scanner noise during the imaging process. Most participants (95.1%, 97/102) cooperated with scanning after verbal guidance and parental accompaniment. For 5 children (3 ICPP, 2 non-ICPP) with anxiety or poor compliance, oral chloral hydrate (50 mg/kg) was used for sedation, with continuous pediatrician monitoring during/after scanning. No sedation-related adverse events (e.g., respiratory depression, vomiting) were observed.

Initial routine cranial MRI scans were conducted to exclude potential structural abnormalities. The scanning sequences included T1-weighted imaging (T1WI), T2-weighted imaging (T2WI), fluid-attenuated inversion recovery (FLAIR), and diffusion-weighted imaging (DWI). Detailed parameters for axial images were as follows: T1WI (fast inversion recovery sequence): repetition time (TR) = 2000 ms, echo time (TE) = 20 ms; T2WI (fast gradient echo sequence): TR = 3500 ms, TE = 80 ms; T2-FLAIR: TR = 8000 ms, TE = 125 ms. All axial scans had a field of view (FOV) of 230 mm × 191 mm × 143 mm, slice thickness of 5 mm, interslice gap of 1 mm, and a total of 20 slices acquired.

Participants meeting inclusion criteria subsequently underwent high-resolution three-dimensional T1-weighted structural imaging (3D-T1WI) using a turbo field echo (TFE) sequence. The imaging parameters were as follows: TR = 7.4 ms, TE = 3.8 ms, slice thickness = 1 mm, no interslice gap, 260 contiguous slices; FOV = 250 mm × 250 mm × 156 mm; matrix size = 228 × 227; number of excitations (NEX) = 1; flip angle = 8°; voxel size = 1.1 mm × 1.1 mm × 1.1 mm. The total acquisition time for the 3D-T1WI sequence was 4 minutes and 16 seconds.

### 2.4 MRI data processing and analysis

#### 2.4.1 VBM analysis

VBM preprocessing and analysis were performed using SPM12 (http://www.fil.ion.ucl.ac.uk/spm/software/spm12) and the CAT12 toolbox (http://www.neuro.uni-jena.de/cat) within MATLAB R2021b (https://www.mathworks.com/products/matlab.html). Processing steps included: (1) DICOM-to-NIFTI conversion: All T1-weighted images were converted from DICOM to NIFTI format using MRIcron’s dcm2nii tool. Scans with motion artifacts, poor contrast, or incomplete coverage were excluded following visual quality control. (2) anterior commissure-posterior commissure AC-PC Reorientation: native T1 images were manually reoriented to align the AC-PC plane with axial slices. (3) Segmentation: CAT12’s default pipeline segmented images into gray matter (GM), white matter (WM), and cerebrospinal fluid (CSF) while performing intensity inhomogeneity correction. Total intracranial volume (TIV), GM volume, WM volume, and CSF volume were extracted. (4) Spatial normalization: segmented GM/WM images underwent affine registration followed by high-dimensional DARTEL normalization to MNI space (ICBM 152 template) with Jacobian determinant modulation to preserve tissue volume information. (5) Spatial smoothing: Normalized-modulated GM images were smoothed with a 15-mm FWHM isotropic gaussian kernel to improve signal-to-noise ratio and accommodate intersubject anatomical variability. (6) Extraction of gray matter volume from significant clusters: Following the identification of significant clusters corrected for false discovery rate (FDR), gray matter volume values were extracted using the ‘Cluster Mask Extraction Tool’integrated in CAT12. For each significant cluster (e.g., the right precentral gyrus), a binary mask was generated to delineate the cluster boundary. The mean gray matter volume within this mask was then calculated for each participant. This approach was adopted to avoid potential biases associated with single-voxel extraction (e.g., variability in peak voxel localization across subjects).

#### 2.4.2 SBM analysis

SBM processing utilized the same software environment (SPM12/CAT12/MATLAB R2021b), with key surface-specific steps:(1) Cortical surface reconstruction:central cortical surfaces were reconstructed from T1 images using the projection-based thickness (PBT) method in CAT12. Topological corrections (e.g., spherical harmonics) were applied to ensure manifold integrity. (2) Spatial normalization: individual surfaces were non-linearly registered to the FreeSurfer fsaverage template via spherical mapping to minimize inter-subject folding variability.(3) Cortical thickness mapping: vertex-wise cortical thickness (distance from pial to white matter surface) was calculated and resampled to the template surface. (4) Spatial smoothing: thickness maps were smoothed on the surface using a 15-mm FWHM gaussian kernel (CAT12 default) to reduce gyral variability. Significance was determined by vertex-level uncorrected *p* < 0.001, FDR-corrected *p* < 0.05, and minimum cluster size (50 vertices)—consistent with VBM’s statistical logic, adapted to surface analysis units.(5) Extraction of cortical thickness from significant surface regions: For regions with significant cortical thickness differences (e.g., the right precentral gyrus), vertex-level cortical thickness values were extracted via the’Surface Vertex Averaging Tool’in CAT12. All vertices within the FDR-corrected significant surface region were selected, and the mean cortical thickness across these vertices was computed for each participant.

#### 2.4.3 Complementarity of VBM and SBM

VBM and SBM are two complementary analytical approaches for structural MRI: VBM evaluates gray matter volume at the voxel level (capturing regional tissue volume changes), while SBM focuses on cortical thickness at the surface vertex level (reflecting the distance between the pial and white matter surfaces). This combination allows us to assess brain structural alterations from two distinct dimensions (volume vs. cortical thickness), avoiding biases from a single analytical perspective.

Both methods use identical FDR correction (*p* < 0.05) and uncorrected *p* < 0.001; only cluster size differs (VBM > 80 voxels, SBM > 50 vertices) to match their analysis units (voxel/vertex).

### 2.5 Statistical analysis

Statistical analyses were performed using SPSS 24.0 (IBM, USA). Demographic and clinical data were assessed for normality using the Shapiro-Wilk test. Normally distributed continuous variables were expressed as mean ± standard deviation ( $\bar{\text{x}}$ ± SD) and compared using independent samples t-tests, while non-normally distributed data were presented as median (interquartile range) [M (Q1,Q3)] and analyzed using Mann-Whitney U tests. Categorical variables were reported as frequencies or percentages and compared using chi-square or Fisher’s exact tests as appropriate. The significance threshold was set at *p* < 0.05.

For neuroimaging analyses, two-sample t-tests were conducted in SPM12 to compare gray matter volume or cortical thickness between groups, with age, height, weight, and total intracranial volume (TIV) included as covariates. Statistical results were corrected for multiple comparisons using FDR, with significance thresholds set at voxel-level *p* < 0.001 and FDR-corrected *p* < 0.05. Results were visualized using xjView 9.6.

Further, correlation analyses were restricted to the ICPP group to investigate associations between structural values (i.e., gray matter volume of VBM-identified significant regions and cortical thickness of SBM-identified significant regions) and clinical measures. Prior to correlation testing, the normality of all variables (including both structural values and clinical measures) was reassessed using the Shapiro-Wilk test. Pearson correlation was employed for pairs of variables where both were normally distributed, while Spearman correlation was used for pairs involving at least one non-normally distributed variable. All correlation analyses were adjusted for covariates including chronological age, standing height, body weight, and TIV. Statistical results were corrected for multiple comparisons using FDR, with significance thresholds set at FDR-corrected *p* < 0.05. Correlation strength was categorized based on the absolute value of the correlation coefficient (|*r*|): 0.7–1.0 (strong), 0.4–0.7 (moderate), and < 0.4 (weak).

## 3 Results

### 3.1 Demographics and characteristics

**Table 1** presents the demographic and clinical profiles of the patients. Chronologic age, height, and weight were normally distributed (mean ± SD); other indicators were non-normal (median[P25,P75]) (**Table 1**). As shown in **Table 1**, ten variables exhibited statistically significant differences between the ICPP and non-ICPP groups (*p* < 0.05), including height, weight, baseline levels of LH and FSH, LH peak value, LH peak/FSH peak ratio, bone age, left and right ovarian volumes, and uterine volume. The ICPP group exhibited significantly greater height, weight, sex hormone levels, bone age, and reproductive organ volumes compared to the non-ICPP group (*p* < 0.05), indicating an accelerated pattern of physical growth and reproductive system development in girls with ICPP. No significant differences were observed between the ICPP and control groups in terms of intelligence, behavioral, and emotional scale scores, suggesting that premature activation of the HPG axis may not have a noticeable short-term impact on cognition, behavior, or emotions. However, the potential long-term effects warrant further follow-up and investigation.

**Table 1:** Demographic and clinical characteristics.

Indicators	ICPP group (n = 52)	Non-ICPP group (n = 50)	Statistic	*p*
General measures				
CA (year)	6.56 ± 1.06	6.64 ± 1.21	1.443 ^a^	0.183
Height (cm)	125.73 ± 6.50	118.11 ± 6.81	3.447 ^a^	<0.001
Weight (kg)	28.16 ± 5.58	24.53 ± 5.43	3.753 ^a^	<0.001
BMI (kg/m^2^)	18.46 (16.14, 19.07)	17.58 (16.06, 18.6)	1.537 ^c^	0.127
Clinical manifestation				
Breast development	44 (85%)	41 (82%)	1.814 ^b^	0.084
Vaginal bleeding	20 (38%)	17 (34%)	1.343 ^b^	0.097
Laboratory indicators				
Basal LH (mIU/ml)	0.25 (0.13, 0.44)	0.11 (0.11, 0.13)	2.045 ^c^	<0.001
Basal FSH (mIU/m)	2.56 (1.75, 3.65)	1.78 (1.36, 2.56)	3.230 ^c^	0.002
Peak LH (mIU/ml)	10.50 (9.23, 12.54)	3.45 (2.45, 4.79)	8.154 ^c^	<0.001
Peak FSH (mIU/ml)	11.28 (9.53, 12.71)	11.45 (9.45, 13.38)	1.267 ^c^	0.087
Peak LH/Peak FSH	0.95 (0.88, 1.13)	0.27 (0.22, 0.38)	9.237 ^c^	<0.001
E2 (pmol/L)	78.24 (72.60, 81.09)	74.60 (72.33, 81.15)	2.125 ^c^	0.055
Imaging features				
BA (years)	9.1 (8.54, 9.57)	8.45 (7.58, 9.32)	3.254 ^c^	0.001
Left ovarian volume (cm^3^)	1.32 (1.13, 1.87)	0.89 (0.69, 1.77)	3.214 ^c^	<0.001
Right ovarian volume (cm^3^)	1.35 (0.89, 1.78)	0.89 (0.60, 1.76)	3.234 ^a^	<0.001
Uterine volume (cm^3^)	2.76 (2.30, 3.68)	2.26 (1.76, 2.78)	2.239 ^c^	0.003
Scale				
WISC-CR (IQ) score	103.45 ± 11.36	103.54 ± 12.87	1.564 ^a^	0.675
CBCL score	7.45 ± 5.20	7.34 ± 5.76	1.578 ^a^	0.897
HAMA score	5.67 ± 1.54	5.87 ± 1.08	1.854 ^a^	0.654

n, number of patients; ICPP, idiopathic central precocious puberty; CA, chronologic age; BMI, body mass index; LH, luteinizing hormone; FSH, follicle-stimulation hormone; E2, estradiol; BA, bone age; WISC-CR, wechsler intelligence scale for children-revised chinese revision; CBCL, child behavior checklist; HAMA, hamilton anxiety rating scale; ^a^two-sample t-test for normally distributed data; ^b^chi-square test for classifying categorical variables; ^c^mann-whitney u-test for nonnormally distributed data; The results were considered statistically significant at *p* < 0.05.

### 3.2 Whole-brain volume analysis results

Compared to the non-ICPP group, the ICPP group showed increased total intracranial volume, white matter volume, and cerebrospinal fluid volume, along with decreased gray matter volume (*p* < 0.05) (see **Table 2**).

**Table 2:** Comparison of whole brain structure volume between ICPP group and non-ICPP group.

Group	TIV (cm^3^)	GMV (cm^3^)	WMV (cm^3^)	CSFV (cm^3^)
ICPP group	1367.63 ± 90.57	668.72 ± 24.57	454.65 ± 21.79	245.42 ± 30.32
Non-ICPP group	1323.27 ± 99.90	712.23 ± 25.7	421.08 ± 23.04	202.78 ± 26.84
t	2.538	-2.038	2.203	2.533
*p*	0.013*	0.012*	0.028*	0.011*

TIV, total intracranial volume; GMV, gray matter volume; WMV, white matter volume; CSFV, cerebrospinal fluid volume. Statistical significance was set at *p<0.05.

### 3.3 VBM analysis results

**Figure 3:**
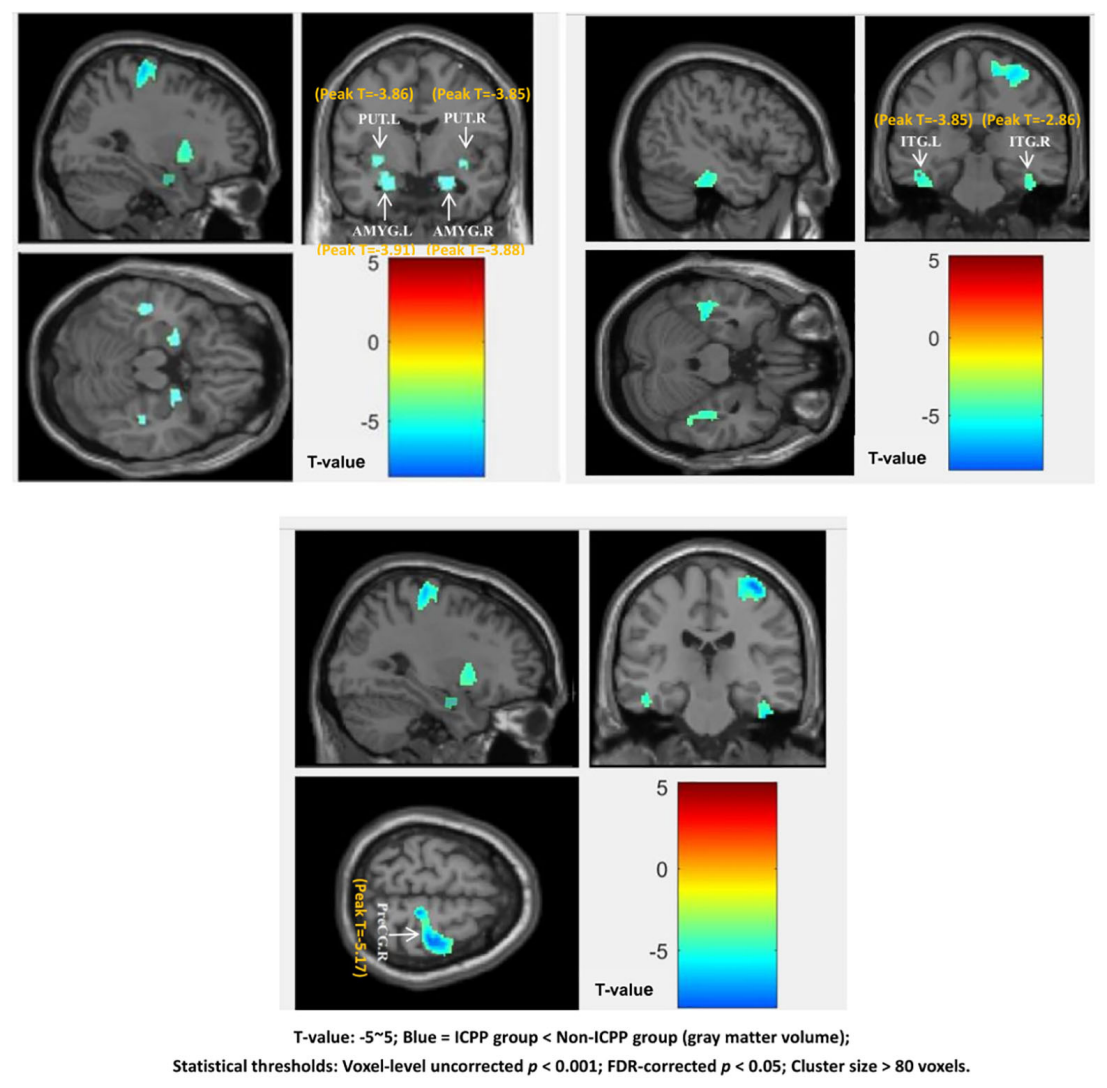
Brain regions showing significant differences in grey matter volume between the ICPP and non-ICPP group. PreCG.R, right precental gyrus; AMYG.L, left amygdala; AMYG.R, right amygdala; ITG.L, left inferior temporal gyrus; ITG.R, right inferior temporal gyrus; PUT.L, left putamen; PUT.R, right putamen.The color bar on the right side of the figure indicates the range of T-values from -5 to 5, where negative T-values represent reduced grey matter volume in the ICPP group compared to the non-ICPP group (the darker the blue, the more significant the reduction). The peak T-value of significant clusters ranges from -5.17 (right precentral gyrus, cluster 1) to -2.86 (right inferior temporal gyrus, cluster 5). Statistical thresholds: voxel-level uncorrected *p*<0.001, FDR-corrected *p*<0.05, cluster size>80 voxels.”.

VBM analysis revealed that, compared to the non-ICPP group, the ICPP group exhibited reduced gray matter volume in multiple brain regions, including the right precentral gyrus, bilateral amygdala, bilateral inferior temporal gyrus, and bilateral putamen (*p* < 0.05, false discovery rate [FDR] corrected) (see **Figure 3**, **Table 3**). Cluster 5 (ITG.R, T=-2.86) is significant as it meets VBM’s criteria: uncorrected *p* < 0.001, FDR-corrected *p* < 0.05, and cluster size=122 voxels (>80 voxels).

**Table 3:** Brain regions showing significant differences in grey matter volume between the ICPP and non-ICPP groups.

Regions	Voxel number	Peak MNI			Peak T	*p*
		X	Y	Z		
Cluster 1						
PreCG.R	406	34.5	-24	67.5	-5.17	<0.05
Cluster 2						
AMYG.L	155	-24	-4	-16	-3.91	<0.05
Cluster 3						
AMYG.R	146	23.5	-7.5	-16	-3.88	<0.05
Cluster 4						
ITG.L	160	-36	-30	-24.5	-3.85	<0.05
Cluster 5						
ILG.R	122	70.5	-46.5	-15	-2.86	<0.05
Cluster 6						
PUT.L	101	-10.5	7.5	-10.5	-3.86	<0.05
Cluster 7						
PUT.R	85	34.5	1.5	-6	-3.85	<0.05

PreCG.R, right precental gyrus; AMYG.L, left amygdala; AMYG.R, right amygdala; ITG.L, left inferior temporal gyrus; ITG.R, right inferior temporal gyrus; PUT.L, left putamen; PUT.R, right putamen; MNI, montreal neurological institute. The statistical significance level was set as *p* < 0.05 after FDR correction.

### 3.4 SBM analysis results

**Figure 4:**
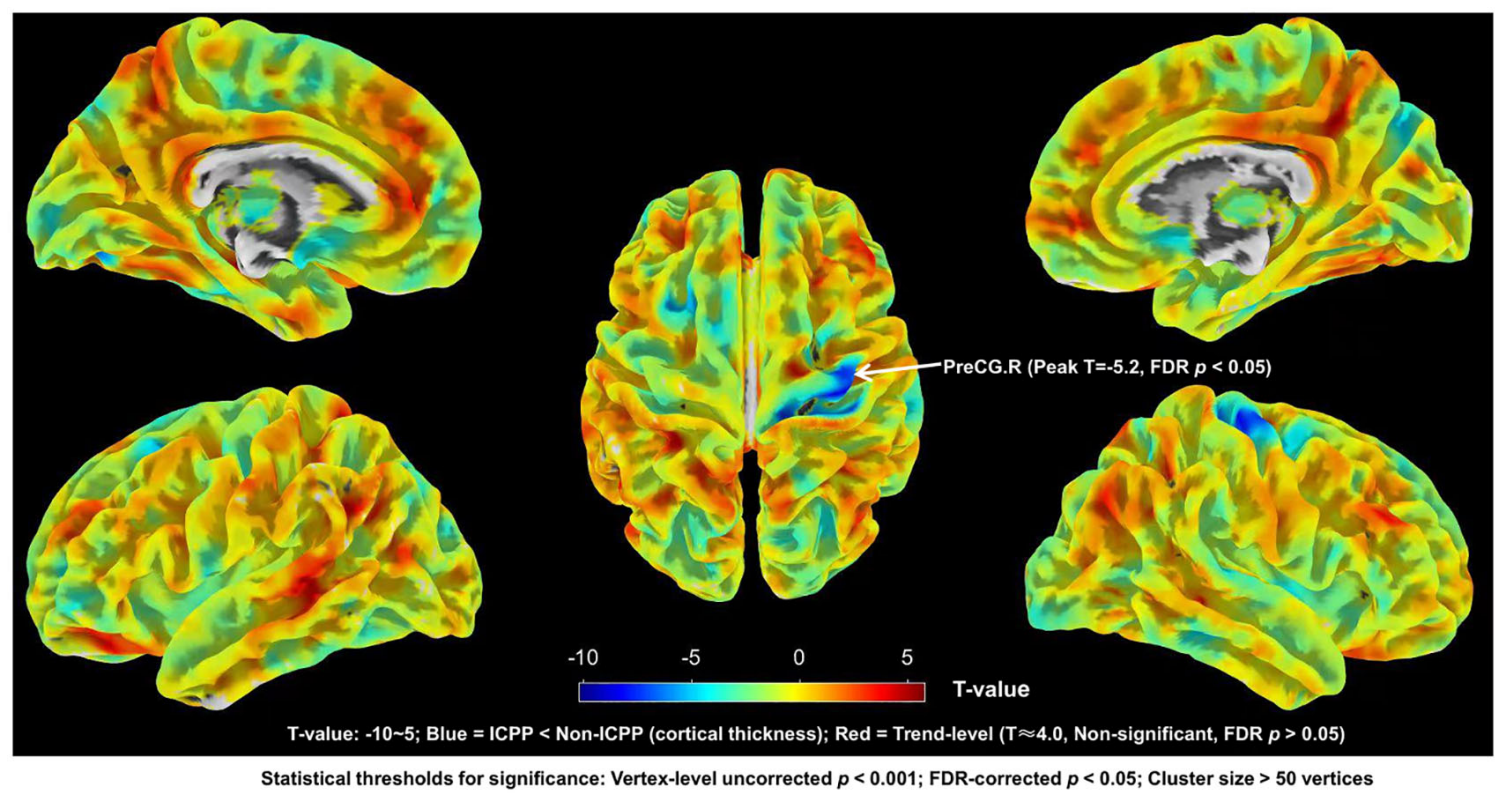
Brain regions with significant differences in cortical thickness between ICPP group and non-ICPP group. ICPP, idiopathic central precocious puberty; non-ICPP, non-idiopathic central precocious puberty. The color bar on the right side of the figure indicates the range of T-values from -10 to 5: negative T-values (blue regions) represent reduced cortical thickness in the ICPP group compared to the non-ICPP group (peak T-value = -5.2 for the right precentral gyrus, the only significant cluster), while positive T-values (red regions, T≈4.0) represent trend-level increased cortical thickness (not significant, as FDR-corrected *p*>0.05 or cluster size<50 vertices). Statistical thresholds for significant regions: vertex-level uncorrected *p*<0.001, FDR-corrected *p*<0.05, cluster size>50 vertices.

SBM results showed that, compared to the non-ICPP group, the ICPP group showed significantly thinner cortex in the right precentral gyrus (FDR-corrected, *p* < 0.05) (see **Figure 4**). Red regions (T≈4) in **Figure 3** are trend-level (FDR-corrected *p* > 0.05 or small cluster size) and not significant; only the right precentral gyrus meets all criteria.

### 3.5 Correlation analysis results

**Figure 5:**
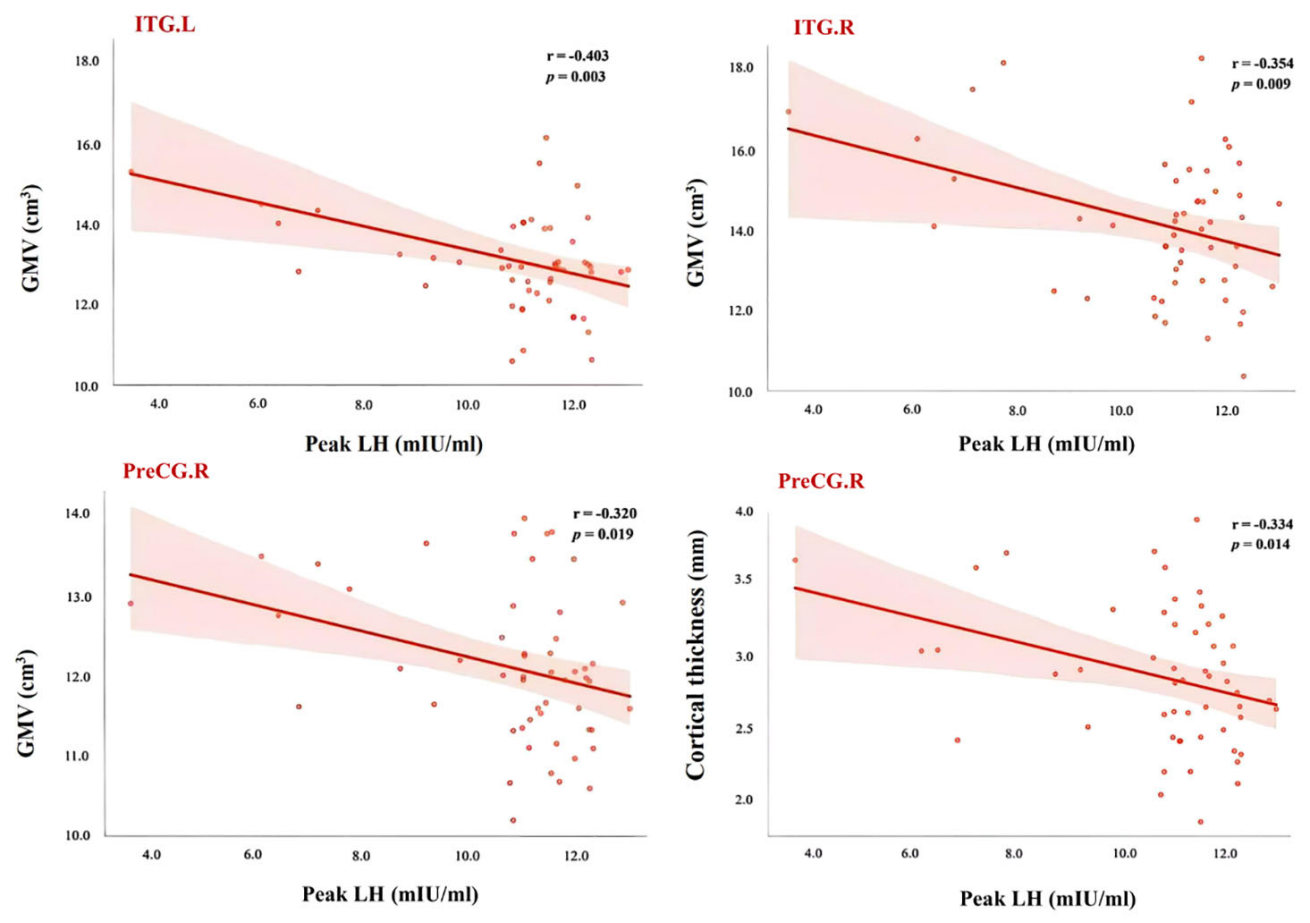
Scatter plot of correlation between LH peak and brain areas showed significant differences between ICPP group and non-ICPP group. PreCG.R, right precental gyrus; ITG.L, left inferior temporal gyrus; ITG.R, right inferior temporal gyrus; LH, luteinizing hormone; GMV, gray matter volume; The statistical significance level was set as *p* < 0.05 after FDR correction.

In this study, correlation analyses were conducted between the gray matter volumes or cortical thickness of brain regions showing significant group differences and the clinical measures within the ICPP group. The results indicated that gray matter volumes of the left and right inferior temporal gyrus were negatively correlated with LH peak levels (left: r = -0.403, FDR-corrected *p* = 0.003; right: r = -0.354, FDR-corrected *p* = 0.009). Additionally, the gray matter volume of the right precentral gyrus also showed a negative correlation with LH peak levels (r = -0.320, FDR-corrected *p* = 0.019). Regarding cortical thickness, the thickness of the right precentral gyrus was likewise negatively correlated with LH peak levels (r = -0.334, FDR-corrected *p* = 0.014) (see **Figure 5**).

Non-significant associations (FDR-corrected *p* > 0.05) included: (1) Gray matter volume of the left amygdala vs. peak LH (r = -0.21, uncorrected *p* = 0.12); (2) Gray matter volume of the right putamen vs. peak FSH (r = -0.18, uncorrected *p* = 0.18); (3) Cortical thickness of the right precentral gyrus vs. E2 (r = -0.15, uncorrected *p* = 0.25). These associations showed weak negative trends but did not reach statistical significance, possibly due to limited statistical power (n = 52 in the ICPP group). Larger-sample studies are needed to validate these potential trends.

## 4 Discussion

To my knowledge, this study is the first to investigate brain structural changes in girls with ICPP using both VBM and SBM. The results showed that, compared to the non-ICPP group, the ICPP group exhibited increased total intracranial volume, white matter volume, cerebrospinal fluid volume, along with reduced gray matter volume. These changes may reflect the influence of hormonal fluctuations on brain structural development in ICPP patients. Previous studies have confirmed multiple mechanisms through which sex hormones affect brain structure, including neurogenesis, programmed cell death, dendritic arborization, and synaptic pruning (16, 25). The reduction in total gray matter volume in girls with ICPP may indicate impaired neuronal development due to hormonal alterations. The increase in white matter volume may be associated with enhanced or restructured neural connectivity. The enlarged CSF volume could be related to the reduction in gray matter, potentially reflecting a sparsification or remodeling of brain architecture.

Using VBM and SBM analyses, this study further revealed significant differences in gray matter volume and cortical thickness between the ICPP and non-ICPP groups across multiple brain regions. VBM results showed decreased gray matter volume in the ICPP group in several areas, including the right precentral gyrus, bilateral amygdalae, bilateral inferior temporal gyrus, and bilateral putamen. SBM analysis revealed reduced cortical thickness in the right precentral gyrus in the ICPP group compared to the non-ICPP group. These regions are involved in complex functions such as motor control, emotional regulation, social cognition, and behavioral decision-making, suggesting that their structural alterations may provide a neurobiological basis for the behavioral abnormalities observed in ICPP patients. Specifically, the precentral gyrus, a critical component of the frontal lobe, plays a major role in motor function and executive control (26). The observed reductions in gray matter volume and cortical thickness in this region may impair motor coordination and higher-order cognitive functions in girls with ICPP. The amygdala, a central structure in the limbic system, is essential for emotional processing, affective memory, fear responses, and reward mechanisms (27, 28). The reduced gray matter volume in the bilateral amygdalae observed in this study may reflect diminished emotional regulation capacity, consistent with previous findings of emotional instability in girls with ICPP (1, 16). The inferior temporal gyrus, a key region of the temporal lobe, is involved in visual processing, object recognition, face recognition, and both emotional and social cognition (29, 30). The reduced gray matter volume in this region suggests that premature activation of the HPG axis may disrupt its normal development, potentially impairing social cognition and emotional regulation in ICPP patients. Additionally, the putamen, an important structure within the basal ganglia, plays a significant role in motor control, emotional regulation, and reward processing (31). The reduced gray matter volume in the bilateral putamen observed in the ICPP group may affect the function of neural circuits related to emotion, motivation, and behavioral regulation. Taken together, these structural alterations in brain regions closely associated with emotional responses, social interaction, reward processing, and behavioral control provide important neuroanatomical evidence for understanding the neurobehavioral abnormalities in girls with ICPP.

Correlation analyses further revealed significant negative associations between peak luteinizing hormone (LH) levels and gray matter volume in the bilateral inferior temporal gyri and right precentral gyrus, as well as cortical thickness in the right precentral gyrus. Peak LH serves as a key biomarker of gonadal axis activation and pubertal onset. These findings indicate an observational association between elevated LH and reduced gray matter volume in regions involved in motor control and social cognition. One potential interpretive framework is that elevated LH could be linked to accelerated synaptic pruning—though this mechanistic pathway remains speculative and requires validation in future experimental studies. This offers direct neuroimaging evidence supporting the modulatory effects of sex hormones on brain development in ICPP girls.

Our results are consistent with and expand upon previous research. For example, Yoshii et al. (15) used SBM to compare brain structural differences between 15 girls with ICPP and 13 healthy controls, finding increased cortical thickness in the right precuneus of the ICPP group. Yang et al. (16) employed SBM to investigate brain structural differences between 28 ICPP girls and 37 non-ICPP girls, reporting cortical thinning in the right middle frontal gyrus in the ICPP group and a correlation between E2 levels and cortical thickness. These studies mainly focused on regional cortical thickness changes. In contrast, the novelty of our study lies in the combined use of SBM and VBM, which revealed not only cortical thinning but also reduced gray matter volume in multiple key brain regions in the ICPP group, particularly in the precentral gyrus, bilateral amygdalae, inferior temporal gyri, and putamen. These areas are closely associated with motor control, emotional regulation, and social cognition, suggesting that ICPP may have potential impacts on motor functions and social behaviors in affected children. Moreover, Chen et al. (17) emphasized a negative correlation between follicle-stimulating hormone (FSH) levels and gray matter volume in the left insula, highlighting the influence of sex hormones on affective and cognitive networks. Our study further enriches this field by combining VBM and SBM techniques, revealing cortical thinning alongside gray matter volume reductions in several brain regions within the ICPP group, with these changes significantly negatively correlated with peak LH levels. This finding not only supports the regulatory role of sex hormones in brain development but also provides the first neuroimaging evidence linking ICPP with alterations in brain regions related to motor control. Unlike Chen et al. (17), who focused on FSH and insular gray matter, our study identified a negative correlation between peak LH levels and gray matter volume in the precentral gyrus, further elucidating how premature activation of the HPG axis affects brain maturation. Additionally, while previous studies predominantly employed a single analytical approach for structural MRI, our combined use of VBM and SBM offers a more comprehensive structural neuroimaging perspective, deepening the understanding of brain structural development patterns in children with ICPP. VBM uniquely identified reduced gray matter volume in the bilateral amygdala, inferior temporal gyrus, and putamen—regions not reported in prior single-modality ICPP studies; SBM complemented this by confirming cortical thinning in the right precentral gyrus (a region also found to have gray matter reduction via VBM), validating overlapping structural abnormalities; Together, they uncovered both overlapping (right precentral gyrus: volume+thickness) and region-specific (amygdala/putamen: only volume) changes, which single methods (e.g., SBM alone in Yoshii et al., 2023 (15), VBM alone in Chen et al., 2019 (17)) failed to capture.

From a clinical perspective, the findings of this study have important implications for the management of children with ICPP. First, the results highlight the need for increased attention to the neurodevelopmental outcomes of children with ICPP, particularly regarding potential impacts on motor control, emotional regulation, and social cognition. This provides new insights for clinical management—for example, incorporating neuropsychological assessments and neuroimaging monitoring during follow-up could offer a more comprehensive evaluation of long-term neurodevelopmental trajectories in this population. Second, the study identifies subtle yet significant structural alterations in brain regions previously linked to motor function (e.g., precentral gyrus), emotional regulation (e.g., amygdala), and social cognition (e.g., inferior temporal gyrus) among ICPP children. Notably, these findings reflect anatomical changes in functionally relevant regions, rather than direct measurements of motor, emotional, or cognitive function itself. These findings suggest the importance of enhanced clinical follow-up focusing on these domains, which may help to identify early warning signs of potential emotional or behavioral issues.

While MRI is costly and may require sedation in young children, its use in this study was justified: it excluded intracranial anomalies (a prerequisite for ICPP diagnosis) and controlled for CNS confounders in the non-ICPP group. The low sedation rate (4.9%, 5/102) and absence of adverse events minimized risks. Future studies could explore selective MRI use (e.g., only for cases with abnormal clinical signs) to balance accuracy and cost-effectiveness in resource-limited settings.

This study has several limitations. First, it focused solely on structural brain alterations in girls with ICPP and did not explore their impact on brain function. Future studies could incorporate functional MRI to further assess the neural network activity patterns in ICPP children, thereby providing a more comprehensive understanding of how precocious puberty affects brain functional organization. Second, due to its cross-sectional design, this study cannot determine whether the observed structural brain changes are a direct consequence of premature activation of the HPG axis or the result of interactions with other potential factors such as genetic or environmental influences. Longitudinal studies are needed to track the long-term neurodevelopmental trajectories of children with ICPP and to clarify whether these early structural alterations have lasting effects on cognitive and emotional functions into adulthood. These improvements would contribute to a deeper understanding of the complex impact of ICPP on neurodevelopment and its underlying mechanisms.

## 5 Conclusions

Based on VBM and SBM techniques, this study effectively detected brain structural changes and remodeling in key regions (precentral gyrus, amygdala, inferior temporal gyrus, and putamen) in girls with ICPP. These structural alterations may serve as potential neuroimaging biomarkers for ICPP and provide new evidence for understanding the neural mechanisms underlying its impact on brain structure.

## Data Availability

The raw data supporting the conclusions of this article will be made available by the authors, without undue reservation.
